# Impact of Thermal Treatment on the Starch-Protein Interplay in Red Lentils: Connecting Molecular Features and Rheological Properties

**DOI:** 10.3390/molecules27041266

**Published:** 2022-02-14

**Authors:** Andrea Bresciani, Davide Emide, Francesca Saitta, Dimitrios Fessas, Stefania Iametti, Alberto Barbiroli, Alessandra Marti

**Affiliations:** Department of Food, Environmental and Nutritional Sciences (DeFENS), Università degli Studi di Milano, G. Celoria 2, 20133 Milan, Italy; andrea.bresciani@unimi.it (A.B.); davide.emide@unimi.it (D.E.); francesca.saitta@unimi.it (F.S.); dimitrios.fessas@unimi.it (D.F.); stefania.iametti@unimi.it (S.I.); alessandra.marti@unimi.it (A.M.)

**Keywords:** pulses, legumes, heat treatment, starch, proteins

## Abstract

Thermal treatments are widely applied to gluten-free (GF) flours to change their functionality. Despite the interest in using pulses in GF formulations, the effects of thermal treatment at the molecular level and their relationship with dough rheology have not been fully addressed. Raw and heat-treated red lentils were tested for starch and protein features. Interactions with water were assessed by thermogravimetric analysis and water-holding capacity. Finally, mixing properties were investigated. The thermal treatment of red lentils induced a structural modification of both starch and proteins. In the case of starch, such changes consequently affected the kinetics of gelatinization. Flour treatment increased the temperature required for gelatinization, and led to an increased viscosity during both gelatinization and retrogradation. Regarding proteins, heat treatment promoted the formation of aggregates, mainly stabilized by hydrophobic interactions between (partially) unfolded proteins. Overall, the structural modifications of starch and proteins enhanced the hydration properties of the dough, resulting in increased consistency during mixing.

## 1. Introduction

Pulses are key to the Mediterranean diet pattern [1] due to several health benefits associated with their consumption [2], such as a reduction in the risk of cardiovascular diseases and type 2 diabetes. In addition, pulse consumption is expanding in response to sustainability and food security issues [3]. Despite some differences among countries, most dietary guidelines recommend an adherence to a plant-based diet [4]. Among plant species, pulses are a good source of proteins, in terms of both protein content (17–30%) and amino acid composition, in particular lysine [5]. When combined with cereal proteins, pulses sustainably provide balanced nutrition [6].

Pulses are mainly consumed as grains, but this entails long preparation and cooking times. Moreover, pulses may contain antinutrients such as phytic acid, trypsin inhibitors, and some non-digestible oligosaccharides, which are related, among others, to some digestive disorders [7].

One approach to increase the consumption of pulses is to use them in the form of flour, which can be added to formulations of cereal-based products [8,9]. Specifically, pulses (or their fractions) enhance the nutritional value of gluten-free products due to their high protein content (21–25%) and dietary fiber (12–20%) [10].

The production of gluten-free products, such as bread, cookies, and pasta, requires the use of flours that have been subjected to a thermal treatment able to modify starch properties. Heat-treated flours exhibited a high water-binding capacity at room temperature, thus impacting on the rheological properties of the dough [11,12,13]. Several studies describing the effect of thermal treatments on cereals (mainly rice and corn) are reported in the literature [12]. Since starch properties (including swelling and water-binding capacity) can vary according to processing conditions and starch type [12], there might be a change in the competition for the available water between starch gelatinization and protein denaturation processes during the heat-treatment of pulses. In the case of pulses, the modification of starch and proteins upon processing have seldom been evaluated at the molecular level. As a matter of fact, countless studies underscore that understanding individual molecular events in terms of their impact on each class of macromolecules—and of specific components within each class—may be of great value, not only from the standpoint of science at large but also for when treatment parameters need to be adjusted according to a specific raw material or a specific food.

In current practice, pulses are thermally treated at industrial level on an empirical basis, most often duplicating the same processes and technologies used for cereals. Heat-treated pulses are expected to have increased nutritional properties, with a lower content of anti-nutritional factors [14], and improved technological properties [12]. Nevertheless, to the best of our knowledge, there is no evidence regarding the effect of the above-mentioned process on the functional properties of pulse flour and their relationship with the structural organization of starch and proteins or their interactions.

For this reason, this study primarily aims to investigate the effects of a thermal treatment carried out on an industrial scale, and thus representative of common industrial practice, on the molecular characteristics of starch and proteins in pulses. Red lentils (*Lens culinaris*) have been chosen as the raw material because they are the second-most consumed pulse after chickpeas and because their use in cereal-based products is growing [15,16]. To this aim, one thermal-treated and one non-treated sample from the same commercial batch of red lentils, produced by an Italian mill as an ingredient for food production, were compared. Investigating the relationship between the molecular changes and rheological properties of red lentil dough is the second objective of the present study. Since no studies are available on the effect of thermal treatments on starch and protein features in red lentils, addressing this topic could help to identify ideal raw materials for specific applications and/or to design specific process conditions.

## 2. Results and Discussion

### 2.1. Impact of Heat Treatment on the Color of Red Lentil Flour

Color plays a fundamental role in consumer choice and product acceptability. The heat treatment led to a significant (*p* < 0.001) increase in the degree of brightness (91.1 versus 81.1) and redness (10.5 versus 9.3) of the red lentil flour. These differences were not attributable to differences in flour particle size distribution, which was similar in both samples (data not shown). However, the modest change in overall color (ΔE less than 4) suggests that the overall differences in color might not be perceivable to the human eye [17].

### 2.2. Starch Properties

#### 2.2.1. Heat Treatment Affects Starch Susceptibility to α-Amylase Hydrolysis

An evaluation of the amount of starch susceptible to rapid hydrolysis (i.e., 10 min) by α-amylase was used to provide information on process-related changes to starch: the higher the value of readily hydrolyzable starch, the higher the degree of gelatinization [18]. The thermal treatment applied to red lentils led to a modest but significant (*p* < 0.01) increase in susceptibility to rapid α-amylase hydrolysis. Heat-treated flour showed a slightly higher (+8%) amount of starch that was quickly accessible to hydrolysis compared to the untreated sample (5.4 ± 0.1 versus 5.0 ± 0.1 g/100 g of total starch).

However, it is important to note that the effect of heat treatment is reportedly much greater in cereals, such as rice or corn, than in pulses. For example, heat-treated rice shows a more than 50% increase in the amount of starch susceptible to enzymatic hydrolysis [18]. Pulse starch is characterized by a high amount of amylose (30%) [19,20] that requires high energy for gelatinization [21], a feature that may limit the effects of the thermal treatment of red lentils. Moreover, the low starch content in pulses, together with their high content in protein and fiber, may further contribute to limiting starch gelatinization during heat treatment (likely due to the competition for water between starch and proteins) and, therefore, the susceptibility of starch to rapid α-amylase hydrolysis even after the thermal treatment.

#### 2.2.2. Thermal Analysis Highlights the Modification of Both Starch and Proteins

**Figure 1:**
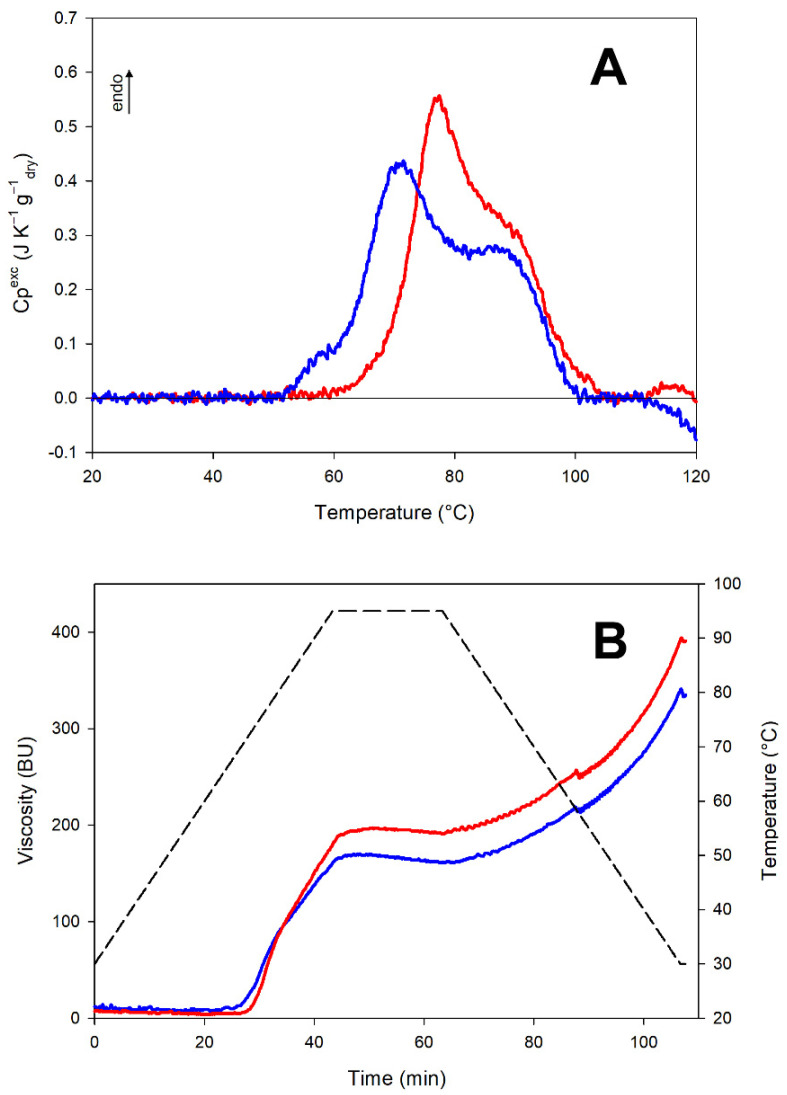
Starch properties. Raw flour (blue line) and heat-treated flour (red line) from red lentils. Panel (**A**): Differential scanning calorimetry (DSC) traces recorded at 60% moisture content. Panel (**B**): Micro Visco-Amylo-Graph traces.

The thermal behavior of raw and heat-treated flours from red lentils was assessed through differential scanning calorimetry (DSC) (Figure 1A). A complex multiphasic endothermic signal was observed in untreated red lentil flour, where the signal mainly reflected the starch gelatinization process. In particular, the main peak (at about 70 °C) corresponds to the starch gelatinization that relies on water immediately available in the system (the higher the water content, the higher the percentage of gelatinized starch in this first step), whereas the second shoulder in the thermograms in Figure 1A (above 80 °C) corresponds to the completion of the process [22]. On the other hand, an initial shoulder is observed in the DSC trace (visible below 60 °C). Such a shoulder is not common for starch gelatinization occurring in DSC experiments carried out at a high moisture level, which instead is generally observed to begin with a very steep increase in Cp^exc^ [22]. This small additional thermal contribution might be ascribed to proteins. Indeed, lentils contain proteins with a distribution of different thermal stabilities [23]. Moreover, the thermal stability of globular proteins in complex systems is moisture-dependent and, in our DSC conditions (i.e., 60% moisture, 2 °C/min), a low-temperature onset of protein denaturation is plausible [24]. The overall enthalpy of these changes corresponds to Δ*H*_raw_ = 10.5 ± 0.5 J/g_dry_.

The DSC thermogram of the flour from treated red lentils reveals substantial differences in the system’s thermal behavior. Specifically, the protein denaturation contribution is no longer detectable, indicating that the treatment led to a sensible protein denaturation. The starch gelatinization onset appears shifted towards higher temperatures: from about 60 °C in untreated flour to 70 °C in the flour from treated materials. Since the gelatinization onset temperature relies heavily on starch composition and structure [22], the observed upshift suggests that the treatment had an impact on the starch granule structure as to make the first penetration of water more difficult. In the case of the treated samples, the overall enthalpy remained the same when measured in the raw flour (Δ*H*_treated_ = 10.5 ± 0.5 J/g_dry_).

The overall picture indicates that the heat treatment of red lentils affected the starch granule structure and its gelatinization properties in terms of onset and possibly its kinetics, but was not able to induce starch gelatinization, as suggested by the similarity of the enthalpy values measured on the treated and untreated samples. Evidence for the absence of significant gelatinization is observed in Figure S1 in the Supplementary Material, showing the absence of starch retrogradation in a treated flour sample kept in the presence of excess water at 4 °C for 48 h, a condition that should allow starch retrogradation [25].

#### 2.2.3. Structural Modifications Lead to Different Pasting Properties

The effect of the thermal treatment on starch pasting and retrogradation properties was assessed by measuring the changes in viscosity upon heating and cooling steps under controlled conditions. As the temperature increased, the viscosity of the system increased because starch (in the presence of excess water) undergoes swelling, followed by the rupture of its granules and loss of original organization (i.e., gelatinization), leading to a decrease in viscosity. As the temperature decreases, the viscosity increases due to amylose reorganization in a more organized structure (i.e., retrogradation).

The pasting profiles of individual samples are reported in Figure 1B. Raw flour showed a significantly lower (*p* < 0.05) pasting temperature than the heat-treated sample (70.6 and 72.9 °C), likely depending on the change in the onset of gelatinization observed in DSC thermograms (Figure 1A). This confirms that the industrial process applied in this study might have resulted in starch and/or starch granule structure reorganization. Indeed, starch in heat-treated flour required a higher temperature to start the gelatinization [26]. Moreover, the heat-treated flour showed a significantly higher (*p* < 0.05) peak viscosity (202.5 ± 7.8 BU) than the raw flour (164.5 ± 7.9 BU). This suggests that the thermal treatment, even if modifications were only minor, made the starch granules more capable of absorbing water during the test, resulting in a higher viscosity due to the higher gelatinization capacity. During cooling, a significant (*p* < 0.05) higher final viscosity was observed in the treated flour (394 ± 12 BU versus 320 ± 21 BU), indicating that the treated material underwent a greater reorganization as a consequence of a greater gelatinization, with no impact on the retrogradation capacity, since no differences in setback values were observed (data not shown).

Our findings are consistent with those reported for yellow pea [27]. In the previous study, a high viscosity was also associated with changes occurring to the protein fraction, that could form a network capable of retaining water and increasing the viscosity of the system. Jiang et al. [28] attributed the higher viscosity of thermally treated pulses to the denaturation of water-soluble proteins. Proteins can have a significant impact on starch pasting properties by forming aggregates through protein–protein interactions [29], by changing the leaching behavior of starch components [30] and increasing water retention [31].

### 2.3. Proteins Features

#### 2.3.1. Heat Treatment Affects Protein Structure and Aggregation

The compactness of the protein structure was studied by investigating their susceptibility to the actions of proteolytic enzymes. In most cases, a low accessibility to proteases is associated with a native and compact structure, whereas increased accessibility is associated with the flexible structure typical of partially unfolded proteins [32]. Proteins in the heat-treated flour showed a significant (*p* < 0.001) increase in susceptibility to tryptic hydrolysis compared to those in the raw sample, releasing a higher amount of TCA-soluble peptides (absorbance at 280 nm of the TCA supernatant was 0.286 ± 0.027 and 0.115 ± 0.025, respectively).

**Figure 2:**
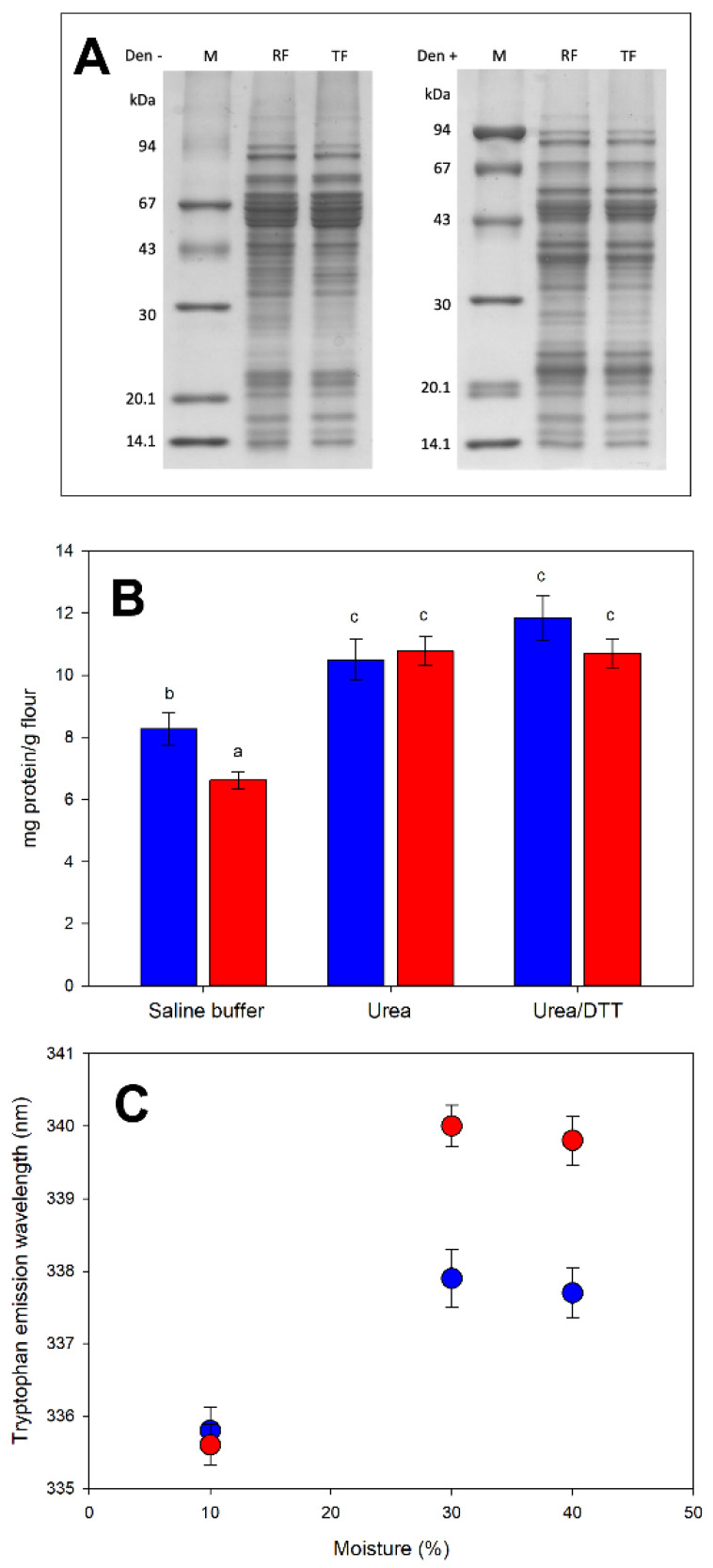
Protein properties. Panel (**A**): SDS-PAGE in non-reducing (Den−, left) and reducing (Den+, right) conditions; M: markers; RF: raw flour; TF: heat-treated flour. Panel (**B**): Protein differential solubility in non-dissociating (saline buffer), dissociating (urea), and reducing (urea/DTT) buffers for raw (blue) and heat-treated (red) flour. Samples marked with the same letter (a, b, c) are not significantly different (*p* < 0.05). Panel (**C**): Tryptophan emission maximum recorded in front-face fluorescence upon kneading raw (blue dots) or heat-treated (red dots) flour with different amounts of water. DTT: dithiothreitol.

The protein pattern of the raw and heat-treated red lentil flour was characterized by sodium dodecyl sulfate polyacrylamide gel electrophoresis (SDS-PAGE). The comparison of the protein patterns obtained in the presence/absence of a disulfide-reducing agent is shown in Figure 2A. No main differences were observed between raw and heat-treated flour in non-reducing conditions (Figure 2A, left), suggesting that the thermal treatment did not induce the formation of covalent aggregates stabilized by disulfide bridges. In this condition, the two main storage protein families, namely the 11S/legumin-like (60 kDa subunit), and the 7S/vicilin-like (50 kDa subunits), are grouped in the upper part of the electrophoretic separation [33]. This observation is confirmed by the addition of 2-ME as a disulfide reductant (Figure 2A, right) that breaks the disulfide bridge inside the 11S/legumin-like subunit and releases its 20 and 40 kDa polypeptides [33].

More insights on the formation and properties of protein aggregates can be obtained by differential solubility approaches [34,35], which rely on the ability of dissociating and reducing agents to sequentially solubilize the proteins in aggregates stabilized by either non-covalent (mainly hydrophobic) or covalent (disulfide bridges) interactions. The number of proteins solubilized in saline buffer (i.e., when the soluble proteins are not involved in aggregates) was lower in heat-treated than in raw flour (Figure 2B), suggesting that the heat treatment promoted the formation of aggregates between somehow unfolded proteins. In the presence of a chaotrope (4 M urea) and a reductant (4 M urea + 10 mM DTT) (i.e., a condition where soluble proteins are involved in non-covalent or covalent aggregates, respectively) the number of solubilized proteins increased, but no significant differences were observed. This observation suggests that aggregates formed during the thermal treatment are mainly stabilized by hydrophobic interactions (and not by covalent interactions). This is consistent with both the SDS-PAGE separation (Figure 2A, where non-covalent interactions cannot be detected, since the test is carried out in denaturing conditions) and the DSC measurements (Figure 1A, where the endothermic contribution related to protein denaturation was no longer visible in the treated flour), suggesting protein denaturation and possible aggregation during the treatment.

The quantification of the accessible -SH groups in saline buffer provided further information on the structural organization and overall compactness of the protein network, as well as on the reactivity of cysteine residues, which are potentially relevant for the formation of intermolecular disulfide bonds in further processing steps. Thermal treatment resulted in a significant (*p* < 0.05) decrease in the accessibility of thiols (1.10 ± 0.16 and 1.62 ± 0.13 μmol -SH/g fluor in heat-treated compared to raw flour, respectively). The thermal treatment influenced the structure of proteins by increasing their compactness, resulting in a lower accessibility of the cysteine thiols that became buried in the protein network. The formation of aggregates and the reduction in thiol accessibility as a consequence of thermal treatments is also a general trait in other gluten-free raw materials, such as rice [36], no matter whether thermal treatments are applied before or during the pasta-making process.

#### 2.3.2. Water Availability in Drive Protein Modifications during Kneading

Tryptophan (intrinsic) front-face fluorescence was used to study structural modifications of proteins induced by kneading, as a function of the water content of the system. Reportedly, solvation allows proteins to achieve the structural flexibility needed to undergo the modifications induced by physical, chemical, or enzymatic treatments [37]. By increasing the water content of the matrix (from 10% up to 40%), a progressive red-shift (toward higher wavelength) of the maximum tryptophan emission is observed (Figure 2C). The red-shift occurs when the chemical environment of the tryptophan side chains becomes more polar, which happens when hydrophobic regions are exposed to water during protein unfolding. Tryptophan exposure was achieved at 30% moisture content, although the red shift in the heat-treated sample was more pronounced than in the raw flour sample (Figure 2C). Increasing the water content by up to 40% resulted in a further evolution of the systems, observable as a slight decrease in the red-shift. This trend reversal was likely due to increased hydrophobic interactions among the regions exposed at a lower water content so that tryptophan side chains were again buried in these “new” hydrophobic surroundings. Overall, the heat-treated sample showed a greater exposure of tryptophan, suggesting greater modification.

Although the tryptophan front-face fluorescence results were extremely informative of the structural changes of proteins, they did not provide a full description of the structural rearrangement of hydrophobic regions in proteins [37,38,39]. Rearrangements of the hydrophobic regions of a protein, either on the surface or in the core, play a key role in establishing interactions among the different components of the matrix (e.g., protein/protein, protein/lipids, protein/starch). The surface hydrophobicity of the protein was studied using the established 1,8-anilino-naphthalene-sulfonate (ANS) fluorescent probe. The probe binds to the exposed hydrophobic patches, becoming fluorescent so that titration with the probe provides information on both the number and the affinity for the probe of hydrophobic regions on the protein surface. Alternatively, it is possible to measure the fluorescence of the probe at fixed probe concentrations, to explore whether other components of the system (water, for instance) or individual processing steps may affect the binding of the probe.

Upon kneading in the presence of 30% water and a slight excess of ANS, heat-treated flour showed a lower fluorescence intensity than raw flour (13.9 ± 1.0 and 20.8 ± 0.9 A.U., respectively; *p* < 0.001), indicating that the protein network of the treated sample has an overall lower accessibility of the surface hydrophobic patches, in line with the presence of aggregates. Increasing moisture by up to 40% allows for the further evolution of structural modifications, here flattening the difference between the two samples (15.4 ± 1.1 and 14.7 ± 0.7 A.U. for raw and heat-treated flour, respectively). Although this behavior requires further investigation, it is possible to hypothesize that the higher moisture allows a higher protein hydration that, in turn, allows for a further protein reorganization during kneading such as was observed in intrinsic fluorescence.

### 2.4. Hydration Properties

#### 2.4.1. Thermogravimetric Analysis Shows Effects on Starch and Protein Phase Separation

**Figure 3:**
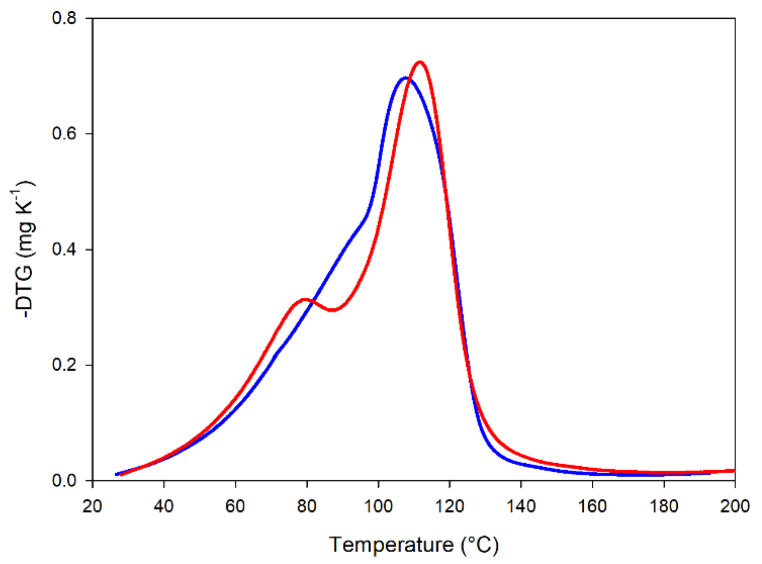
Derivative thermogravimetric (DTG) traces of dough from of raw (blue line) and heat-treated (red line) flours from red lentils at 60% moisture content.

A thermogravimetric analysis (TGA) was carried out to investigate the water distribution in the matrices. Figure 3 reports the derivative thermogravimetric (DTG) traces for raw and treated flours. These traces reflect the rate of water evaporation during heating and represent an indirect index of the strength of water retention in the matrix. The biphasic traces for both the raw and treated materials indicate a non-homogenous water distribution among the starch and protein phases [40]. This phase separation appears more evident in the case of the treated flour. These measurements by themselves do not allow for the assignment of the low and high water retention contributions to starch and/or protein phases. Such a distinction is reported for starch/gluten matrices [40], but no information is available for starch/globular protein systems. A deeper investigation on such a topic is beyond the scope of this phenomenological analysis. It seems safe to say that the treatment produced a variation in water distribution that is coherent with both the modifications of starch granules observed by DSC (Section 2.2.2) and the presence of protein aggregates evidenced by differential solubility experiments (Section 2.3.1), as well as surface hydrophobicity measurements, since protein aggregates are characterized by a decrease in the water-exposed hydrophobic surface (Section 2.3.2).

#### 2.4.2. Thermal Treatment Affects Water-Holding Capacity

The hydration capacity of both raw and heat-treated flours was mostly imputable to their high content of dietary fiber and to the presence of hydrophilic groups in pulse proteins [41]. However, the heat-treated flour showed a slight but significant (*p* < 0.001) increase in the ability to absorb and hold water. In detail, the water-holding capacity was 1.12 ± 0.02 and 0.99 ± 0.02 (g water)/(g flour) for heat-treated and raw flour, respectively. The greater hydration capacity of the heat-treated flour could be related to the more open structure of starch, as also shown by the higher susceptibility to enzymatic hydrolysis. This could favor the hydration of starch granules and lead to a better capacity in the absorption and subsequent retention of water. This behavior was also observed when lentil flour was suspended in water and then heated under control conditions (Figure 1B). The higher viscosity observed in the heat-treated flour was likely due to the greater ability of starch to absorb and bind water. Nevertheless, changes in WHC can also be related to protein denaturation [27,42], as proteins, upon unfolding, expose previously hidden peptide bonds and polar side chains that contribute to their water-holding ability [43].

### 2.5. Mixing Properties

**Figure 4:**
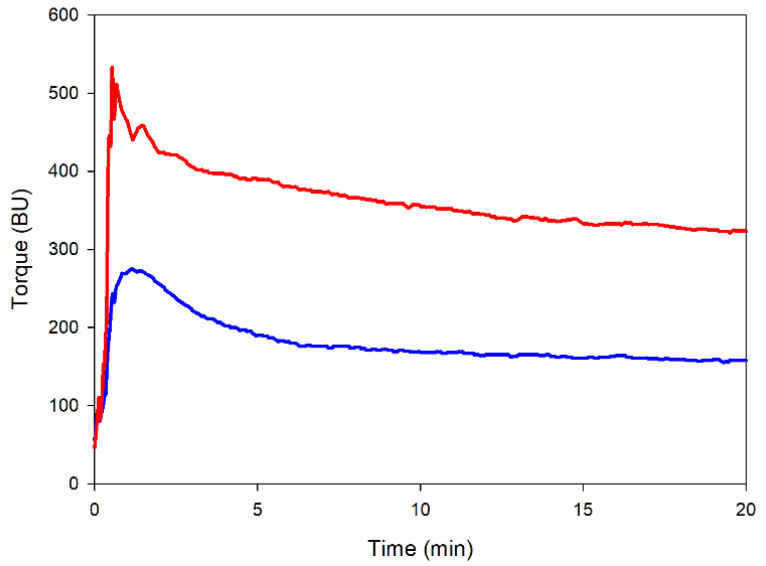
Mixing properties of raw (blue line) and heat-treated (red line) flours from red lentils at 50% hydration level. BU: Brabender Units.

The effect of the heat treatment on the ability of red lentil flour to form a dough was measured at 30 °C and a constant hydration level [44]. The results are reported in Figure 4. The heat-treated samples showed a better ability to form the dough visible from the higher torque values. This result could be due to the better capacity of absorbing water (see Section 2.4.2), which allows the formation of a dough with a higher consistency/torque compared to the raw sample. Similar results were observed for heat-treated cereals such as corn and rice and other types of heat-treated pules (e.g., chickpea) [44].

## 3. Materials and Methods

### 3.1. Samples

Flours from raw and heat-treated red lentils (51% starch; 22% proteins) were provided by an Italian mill. Both samples belonged to the same batch of red lentils. The flours are marketed as ingredients for bread and pasta production.

### 3.2. Color

The color of the flours was measured using a reflectance color meter (CR 210, Minolta Co., Osaka, Japan) equipped with a xenon lamp and a cylinder head to measure the luminosity (L*), redness (a*), and yellowness (b*). Color test was carried out on fifteen replicates. Color difference (ΔE) was calculated as:

(1) $$\Delta E=\sqrt{{\left(L_{1}^{*}-L_{2}^{*}\right)}^{2}+{\left(a_{1}^{*}-a_{2}^{*}\right)}^{2}+{\left(b_{1}^{*}-b_{2}^{*}\right)}^{2}}$$

where 1 and 2 refer to the colorimetric value of untreated and treated flours, respectively.

### 3.3. Starch Properties

#### 3.3.1. Starch Susceptibility to α-Amylase Hydrolysis

This index was assessed as damaged starch (AACC 76-31.01 [45]), which was based on the amount of starch susceptible to rapid hydrolysis by α-amylase. Results were expressed as the total starch content, determined by the standard method AACC 76-13.01 [45], and as mean ± standard deviations of three replicates.

#### 3.3.2. Thermal Properties

The starch gelatinization properties and thermal behavior of the protein fraction in flours were analyzed through a PerkinElmer DSC6 (PerkinElmer, Waltham, MA, USA) working with stainless-steel-sealed pans and experiments from 10 °C to 150 °C at 2 °C/min. An empty pan was used as reference and calibration was carried out with indium as standard. Samples were prepared by directly adding an adequate amount of water to the pans containing about 18 mg of sample to achieve a 60% moisture content. Specifically, for the heat-treated flour, additional DSC measurements were performed for samples that were highly hydrated (72% moisture) and stored at 4 °C for 48 h before measurement in order to highlight possible starch retrogradation. The real final water content for each sample was assessed in the cold pans after the DSC analysis by piercing the pans and desiccating their contents at 105 °C.

Data were analyzed with the dedicated software IFESTOS following procedures reported in previous studies [46]. In brief, the excess heat capacity *C_P_^exc^*(*T*) (J K^−1^ g^−1^_dry matter_), i.e., the difference between the apparent heat capacity *C_P_*(*T*) of the sample and the heat capacity of the pre-transition region, was recorded across the scanned temperature range. Three replicates were performed for each sample and one representative curve for each sample was reported.

#### 3.3.3. Pasting Properties

Pasting properties of pulse flours were evaluated using a Micro Visco-Amylo-Graph, MVAG (Brabender GmbH., Duisburg, Germany) as reported by Bresciani et al. [26]. Briefly, 12 g of flour was dispersed in 100 mL of distilled water, scaling both sample and water weight on a 14% flour moisture basis. The moisture content of flours was evaluated at 130 °C until the sample weight did not change by 1 mg for 60 s in a moisture analyzer (MA 210.R, Radwag; Wagi Elektroniczne, Chorzòw, Poland). The suspensions were subjected to the following temperature profile: heating from 30 up to 95 °C, holding at 95 °C for 20 min and cooling from 95 to 30 °C with a heat/cooling rate of 1.5 °C/min. The viscoamylographic indices (i.e., pasting temperature, maximum viscosity, final viscosity, and setback) were directly calculated by the software (Visograph 2.3.7 Brabender, Duisburg, Germany) provided with the device. The analysis was carried out in duplicate and one representative curve for each sample was reported.

### 3.4. Protein Properties

#### 3.4.1. Protein Susceptibility to Tryptic Hydrolysis

Susceptibility of protein samples to tryptic hydrolysis was studied as described by Iametti et al. [32]. In this study, flour (75 mg) was suspended in 6 mL of 50 mM sodium phosphate, 0.1 M NaCl, pH 7.0, and added to a 150 μL trypsin solution (2 mg/mL in 20 mM sodium acetate pH 4.5) to give an enzyme/protein ratio around 1:50. After incubation at 37 °C for 40 min, the proteolytic reaction was stopped by adding an equal volume of 20% (*w/v*) trichloroacetic acid (TCA). The precipitated undigested proteins were removed by centrifugation (13,000× *g* for 10 min), and soluble peptides were quantified spectrophotometrically by reading the absorbance of the supernatant at 280 nm versus a blank containing only phosphate buffer and TCA. Results were expressed as mean ± standard deviation of three replicates.

#### 3.4.2. Sodium Dodecyl Sulfate-Polyacrylamide Gel Electrophoresis (SDS-PAGE)

For SDS-PAGE, the samples were prepared by suspending 2 mg of flour in 100 μL of water and 100 μL of denaturing buffer (0.125 M Tris-HCl, pH 6.8, 50% glycerol, 1.7% SDS, 0.01% bromophenol blue (*w/v*)), containing 1% (*v/v*) 2-mercaptoethanol (2-ME), when indicated. The suspension was then heated at 100 °C for 10 min. The electrophoretic run was performed at pH 8.8 (0.025 M Tris-HCl, 0.192 M glycine, 0.1% (*w/v*) SDS), in a Miniprotean II cell (Bio-Rad Laboratories, Hercules, CA, USA), and the gels were Coomassie Blue-stained.

#### 3.4.3. Protein Differential Solubility

The solubility of proteins in native and denaturing/reducing conditions was evaluated by differential solubility studies, conducted as reported by Iametti et al. [47]. For these analyses, 150 mg of flour was suspended in 6 mL of saline buffer (50 mM sodium phosphate, 0.1 M NaCl, pH 7.0), containing 4 M urea and 10 mM dithiothreitol (DTT), when required. The suspension was stirred for 90 min at 25 °C and subsequently centrifuged (10,000× *g* for 20 min, 25 °C). The quantification of proteins in the supernatant was carried out by a dye-binding method [48] using a Perkin-Elmer Lambda 2 UV/VIS Spectrometer (Perkin-Elmer Inc., Waltham, MA, USA). Results were expressed as mean ± standard deviations of three replicates.

#### 3.4.4. Quantification of Accessible Thiols

Accessible thiols (-SH) were quantified by exploiting the ability of the reagent 5,5-dithio-bis-nitrobenzoate (DTNB) to react with the exposed -SH groups of cysteine [49], following a procedure reported elsewhere [50]. A 50 mg aliquot of flour was suspended in 6 mL of 50 mM sodium phosphate, 0.1 M NaCl, pH 7.0, containing 0.2 mM DTNB, in the presence/absence of 4 M urea. After 1 h at 25 °C, the suspension was centrifuged (13,000× *g* for 15 min, 20 °C) to remove solid materials, and the absorbance of the supernatant was read at 412 nm against a DTNB blank, in a Perkin-Elmer Lambda 2 UV/VIS Spectrometer (Perkin-Elmer Inc., Waltham, MA, USA). Results are expressed as mean ± standard deviations of three replicates, from two independent measurements.

#### 3.4.5. Front-Face Fluorescence

Intrinsic (tryptophan) fluorescence was assessed by front-face (solid state) measurements, as reported by Bonomi et al. [37]. Solvation studies were performed by adding to 1 g of sample enough water to achieve a final moisture content of between 10% (i.e., the moisture content of flour) and 40%. The mixture was kneaded for 2 min by using a glass stirring rod, and then loaded in the front-face fluorescence cell. Tryptophan fluorescence emission was monitored from 300 to 420 nm (emission slit 2.5 nm), by exciting samples at 280 nm (excitation slit 2.5 nm) with a scan speed of 50 nm/min. All of the measures were conducted at 25 °C in a LS50 B Perkin-Elmer Luminescence Spectrometer (Perkin-Elmer Inc., Waltham, MA, USA). Results were expressed as mean ± standard deviation of three replicates. Protein surface hydrophobicity was determined by using 1,8-anilino-naphthalene-sulfonate (ANS) as the hydrophobic fluorescent probe [37]. Samples were prepared as reported above for the intrinsic fluorescence, except that part of the added water was replaced with an ANS stock solution (5 mM in water) to give a final probe concentration of 0.5 mM in the resulting dough. Fluorescence intensity was monitored at 460 nm (emission slit 2.5 nm) by exciting samples at 390 nm (excitation slit 2.5 nm). Results are expressed as mean ± standard deviation of three replicates.

### 3.5. Hydration Properties

#### 3.5.1. Thermalgravimetric Analysis (TGA)

The water phase separation in the different lentil flours was analyzed by TGA. A Setaram TG-DSC111 (Lyon, France) allowed the simultaneous monitoring of thermal effects (heat flow versus temperature, and mass loss versus temperature). The typical sample mass was 30 mg with 60% of overall water content (as in DSC experiments), and experiments were carried out from 20 °C to 200 °C at 2 °C/min. Each run was repeated twice. One representative profile for each sample is reported. The ratio between the heat flux and the related mass loss rate was found to be equal to the enthalpy of water evaporation temperature range. This check confirmed that the mass loss was substantially related to water evaporation only. All the TG traces were normalized to 100 mg of sample mass. DTG traces, i.e., the temperature derivative of TG traces, were obtained and expressed as mg of lost mass per temperature unit.

#### 3.5.2. Water-Holding Capacity (WHC)

WHC was determined by calculating the amount of water absorbed and retained by the flours. Briefly, 1.0 ± 0.1 g flour was mixed with 10 mL distilled water, vortexed for 30 s, then left for 30 min at 25 °C. Mixtures were centrifuged at 2500× *g* for 20 min, and the supernatant decanted. WHC was calculated as the ratio between grams of water retained per gram of solid. Results were expressed as mean ± standard deviations of three replicates.

### 3.6. Mixing Properties

Mixing properties were measured by using a Farinograph-E^®^ (Brabender GmbH & Co. KG, Duisburg, Germany) equipped with a 50 g mixing bowl and operating at a constant hydration level (i.e., 50% on dry matter) as reported by Bresciani et al. [44]. The analysis was carried out in duplicate and one representative curve for each sample was reported.

### 3.7. Statistics

The data were subjected to t-test (two-tailed distribution) using Statgraphics Plus 5.1 (Statpoint Inc., Warrenton, VA, USA). Differences at *p* < 0.05 (*); *p* < 0.01 (**) and *p* < 0.001 (***) were considered significant. Data from differential solubility were subjected to analysis of variance (one-way ANOVA; *p* < 0.05) by using SigmaPlot version 14.0 (Systat Software Inc., San Jose, CA, USA). When a factor was significantly different, the difference was determined through Tukey’s HSD test.

## 4. Conclusions

This study indicates that red lentils subjected to a heat treatment at industrial level show a peculiar behavior.

The thermal treatment considered in this study promoted structural changes in both starch and proteins in red lentils. This treatment was not able to promote extensive starch gelatinization. At the same time, some molecular change occurs, since the kinetics of gelatinization observed in DSC, as well as the gelatinization, pasting temperatures, and hot viscosity in pasting properties (Figure 1) were affected. Overall, our results suggest that the treatment likely affected the external regions of the starch granules, i.e., those regions that were slightly (but significantly) more susceptible to α-amylase hydrolysis.

The effects of heat treatment on proteins appear to be generally more pronounced. Denaturation leads to the formation of protein aggregates, stabilized mainly through hydrophobic interactions. Disulfide bonds, which are one of the main actors in many thermal coagulation events [51,52], as well as in the formation of the gluten network [53], do not appear to play a significant role in the case of red lentil proteins, at least in these conditions.

Changes in both starch and protein organization affected the hydration and mixing properties of the heat-treated flour. Specifically, the heat-treated flour absorbed more water but showed a lower retention capacity (Figure 3). The higher water-holding capacity, together with the greater protein aggregation tendency/properties, resulted in a dough with higher consistency during mixing (Figure 4).

The practical relevance of these observations is diverse. For instance, the intensity and duration of the thermal process could be increased to promote starch gelatinization above the level reported herein, so to reach an extent that could be desirable for specific gluten-free products and formulations. Another factor that may come into play is the amount of water in the system during thermal treatment, as water availability relates to the rate (and fate) of both protein unfolding and starch gelatinization. A thorough study of these parameters was outside the scope of what is reported here, as this study aimed to characterize materials of common industrial use. In spite of these limitations, this study provides a methodological basis for improving our current understanding of how any modification of the main macromolecules present in a given system may affect its technologically relevant properties, and how processes may be tailored to fulfil specific requirements from the food industry.

## Data Availability

Not available.

## Supplementary Materials

The following supporting information can be downloaded, Figure S1: Differential Scanning Calorimetry (DSC) thermogram obtained for a highly hydrated sample of treated flour from red lentils (black trace) that was stored at 4 °C for 48 h before measuring to evaluate the presence of starch retrogradation (72% humidity; closed pans; 2 °C/min scanning rate). For the sake of comparison, the DSC profile obtained for the treated flour of red lentils (already reported in Figure 1A in the main text) is also shown as a red trace (60% humidity; closed pans; 2 °C/min scanning rate).

## Sample Availability

Not available.
