# Supplementary material for: Impact of Thermal Treatment on the Starch-Protein Interplay in Red Lentils: Connecting Molecular Features and Rheological Properties
Source: Molecules. 2022 Feb 14;27(4):1266. doi: 10.3390/molecules27041266 (PMC8879514; doi:10.3390/molecules27041266)
Supplement: Supplementary file 1 [file molecules-27-01266-s001.zip › molecules-1544375-supplementary.pdf]

Supplementary Material

Impact of thermal treatment on the starch-protein interplay in red lentils: connecting molecular features and rheological properties

Andrea Bresciani[+], Davide Emide[+], Francesca Saitta, Dimitrios Fessas, Stefania Iametti, Alberto Barbiroli*, Alessandra Marti

Department of Food, Environmental and Nutritional Sciences (DeFENS), Università degli Studi di Milano, via G. Celoria 2, 20133 Milan, Italy

[+] these authors contributed equally to this work

*Corresponding author: alberto.barbiroli@unimi.it

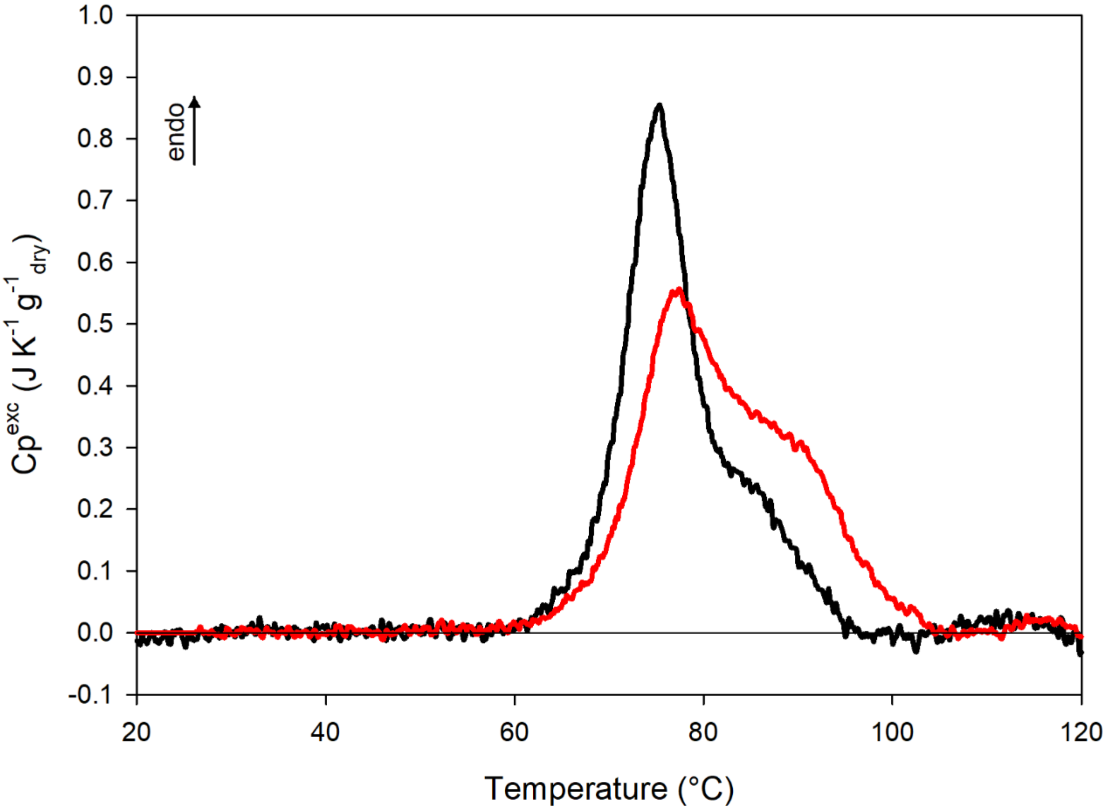

Figure S1. DSC thermogram obtained for a highly hydrated sample of treated flour from red lentils (black trace) that was stored at 4°C for 48 hours before the measurement for evaluating the presence of starch retrogradation (72% humidity; closed pans; 2°C/min scanning rate). For the sake of comparison, the DSC profile obtained for treated flour of red lentils (already reported in Figure 1A in the main text) is also shown as a red trace (60% humidity; closed pans; 2°C/min scanning rate).

Raw and heat treated flours show no differences in their overall gelatinization enthalpy (ΔH_{gel} = 10.5 ± 0.5 J/g_{dry} for both systems).

An additional DSC measurement was carried out on heat treated flour by choosing a different experimental approach as to favour the occurrence of any possible starch retrogradation. In details, a highly hydrated sample (water amount above 70%) was stored at 4°C for 48 hours before the DSC measurement. The DSC thermogram (Figure S1, black trace) show no starch retrogradation despite the favourable experimental conditions. Indeed, if any starch retrogradation occurred, it would have been visible as an endothermic contribution at about 40°C [23].

Although the experimental methods applied were different, it is possible to observe that the water content affects the kinetics of the gelatinization, since the amount of water immediately available for the process is different [22]. Specifically, the higher the water content, the higher the gelatinization rate and the amount of gelatinized starch at the first step (first gelatinization peak). On the other hand, we may notice that the gelatinization onset temperature does not undergo any variation since it only relies on the native starch composition and structure [22]. Moreover, the enthalpy of gelatinization kept the same values at both moisture levels.
